# The effects of smoking, regular drinking, and unhealthy weight on health care utilization in China

**DOI:** 10.1186/s12889-021-12309-z

**Published:** 2021-12-11

**Authors:** Changle Li, Zhengzhong Mao, Caixia Yu

**Affiliations:** 1 School of Health Management, Inner Mongolia Medical University, Hohhot, 010110 China; 2 West China School of Public Health, Sichuan University, Chengdu, 610041 China

**Keywords:** Smoking, Regular drinking, Unhealthy weight, Health care utilization; China

## Abstract

**Background:**

Preventive risk factors such as smoking, drinking, and unhealthy weight have contributed to the accelerated rise in noncommunicable chronic diseases, which are dominant drivers of health care utilization and spending in China. However, few studies have been conducted using a large longitudinal dataset to explore the impact of such preventive risk factors on health care utilization. Therefore, this study aimed to ascertain the effects of smoking, regular drinking, and unhealthy weight on health care utilization in China.

**Methods:**

This research was a longitudinal study using data from five waves of the China Family Panel Studies (CFPS) conducted between 2010 and 2018, and the final sample consisted of 63,260 observations (12,652 participants) across all five waves of data collection. Health care utilization was measured from two perspectives: outpatient utilization and inpatient utilization. Smoking status was categorized as never smoker, former smoker, or current smoker. Unhealthy weight was classified based on the participants’ body mass index. A fixed effects logistic regression model was used for the analysis.

**Results:**

The results of fixed effects logistic regression showed that current and former smokers were approximately 1.9 times and 2.0 times more likely to use outpatient care than those who never smoked, respectively (odds ratio (OR) = 1.88, *p* < 0.05; OR = 2.03, *p* < 0.05). Obese people were approximately 1.3 times more likely to use outpatient care than healthy weight people (OR = 1.26, *p* < 0.05). Moreover, the results show that compared to those who never smoked, for current and former smokers, the odds of being hospitalized increased by 42.2 and 198.2%, respectively (OR = 1.42; *p* < 0.1, OR = 2.98; *p* < 0.05). Compared to healthy weight people, overweight and obese people were also more likely to be hospitalized (OR = 1.11; *p* < 0.1, OR = 1.18; *p* < 0.1, respectively).

**Conclusion:**

Among Chinese adults, current and former smokers were more likely to use outpatient and inpatient care than those who had never smoked. Moreover, compared to healthy weight people, obese people were more likely to use outpatient and inpatient care, and overweight people were more likely to use inpatient care. These results may have important implications that support the government in making health care resource allocation decisions.

## Background

The Chinese population is aging quickly, which is resulting in an increasing prevalence of chronic diseases and disabilities [1]. At the same time, aiming to improve the health care delivery system and the financing of health care, China has achieved near-universal health insurance coverage, with 95% of the population being insured in 2011 [2]. Population aging and improved insurance coverage can increase the likelihood of health care utilization. Therefore, to improve health care quality and reduce health care costs, it is crucial to analyze the health care utilization issues that are present in China [3].

The National Report on Nutrition and Chronic Diseases of the Chinese Population in 2015 showed that the estimated prevalences of current tobacco smoking, regular drinking, and obesity among adults were 26.6, 25.5, and 11.9%, respectively [4]. Preventive risk factors such as smoking, drinking, and unhealthy weight have contributed to the accelerated rise in noncommunicable chronic diseases in China [5]. Chronic diseases are dominant drivers of health care utilization and spending [6], and the economic burden of chronic diseases in China is estimated to reach $7.7 trillion from 2010 to 2030 [7]. Through their links with the subsequent decrease in health status, smoking, drinking, and unhealthy weight impose an enormous cost on society through premature mortality and increased medical costs. Therefore, it is necessary to examine the association of these preventable risk factors with health care utilization and costs.

In the general population, the difficulty of estimating the effect of smoking status on health care utilization has led to conflicting findings. Many studies have reported that current and former smokers show significantly higher odds of outpatient utilization than those who never smoke [8–12]. Interestingly, some studies have found that current smoking has an association with fewer outpatient visits [13–15]. Therefore, more research is necessary to examine the relationship between smoking and outpatient care utilization. The effect of smoking status on inpatient care utilization has been found to be similar across a number of studies; i.e., compared to people who have never smoked, current and former smokers have a higher likelihood of being hospitalized [10, 13, 14, 16, 17].

The findings regarding the effect of alcohol use on health care utilization are mixed. Some studies have shown that problematic alcohol users are more likely to use health care, including outpatient visits, emergency services, and hospitalization, than abstainers [18, 19]. In contrast, many studies have found that alcohol users are less likely to use health care than abstainers [17, 20–23].

Several studies have examined the associations between unhealthy weight and health care utilization. For example, some researchers have found that overweight and obesity are positively associated with primary care utilization [24–26]. Moreover, overweight and obese people are significantly more likely to be hospitalized [27, 28]. Furthermore, underweight patients are more likely to be hospitalized and visit the emergency room compared to patients with a higher body mass index (BMI) [29].

Most studies have reported the effects of smoking, drinking, and unhealthy weight on health care utilization using cross-sectional data. The main estimation methods include logistic regression, Poisson regression, negative binomial regression, and hurdle regression [15, 16, 18, 19, 24, 27]. However, the chosen variables of health-related behaviors are most likely to be considered endogenous in regression equations. One of the most frequent estimation techniques used to address endogeneity bias in cross-sectional data is the instrumental variable technique [30]. Instrumental variables can control for the selection bias that arises due to omitted variables that capture an individual’s decision [31]. Due to the increased availability of longitudinal data, panel data models offer a solution to the endogeneity problem without resorting to instrumental variables [32].

To the best of our knowledge, few studies have been conducted using a large longitudinal dataset to explore the impact of smoking, regular drinking, and unhealthy weight on health care utilization in China [11, 33]. Two earlier studies in China focused on smoking behavior, not drinking, and unhealthy weight. In addition, the target populations of the two studies were rural residents and middle-aged and older adults; thus, their results may not be generalizable to the Chinese population. As the growing number of longitudinal studies in recent years has shown, longitudinal research designs can answer more social research questions in a much more convincing manner than other research designs [34]. Therefore, the objective of this study is to ascertain the effects of smoking, regular drinking, and unhealthy weight on health care utilization in China. This knowledge will allow us to better understand the associations between smoking, regular drinking, unhealthy weight and health care utilization, thus helping health policy decision-makers make health care resource allocation decisions.

## Methods

### Data source

The database used in this study was obtained from the China Family Panel Studies (CFPS), conducted by the Institute of Social Science Survey of Peking University. The CFPS is a general-purpose, nationally representative, longitudinal survey that includes community, family, adult, and child questionnaires. The survey sample was drawn from 25 provinces and their administrative equivalents, thus representing 95% of the Chinese population. A multistage probability proportional to size sampling was used for the survey. The CFPS respondents were followed every 2 years, and the first wave in 2010 covered a sample of 14,960 households with 33,600 adults (above 16 years old) and 8990 children (younger than 16 years old). Four waves of full-sample follow-up surveys in 2012, 2014, 2016, and 2018 covered 13,315 households with 35,720 adults and 8624 children, 13,946 households with 37,147 adults and 8617 children, 14,019 households with 36,892 adults and 8427 children, and 14,218 households with 37,354 adults and 8735 children, respectively. More details about the CFPS are available from Xie and Hu [35].

Since 2012, the CFPS has classified family members into three groups: gene, core, and noncore members. All gene and core members complete full-length questionnaires. However, noncore members only need to complete only abbreviated questionnaires that collect some critical information. Only the adults who responded to the full-length questionnaires in all waves were selected. The successful tracking rates were 80.6, 83.8, 82.0, and 80.8% at the individual level for the 2012, 2014, 2016, and 2018 follow-ups, respectively. Therefore, this study created a five-period balanced panel dataset. After eliminating all cases with missing relevant data, the final analytic sample consisted of 63,260 observations (12,652 participants) across all five waves of data collection.

### Dependent variables

The current study measured health care utilization from two perspectives: outpatient care utilization and inpatient care utilization. Outpatient care utilization was set as a dummy variable that equaled 1 if the individual self-reported an outpatient visit in the previous 2 weeks before the date of the interview and 0 otherwise. The CFPS question that supports this variable is as follows: ‘Did you visit a doctor in the past two weeks?’. Inpatient care utilization was also set as a dummy variable that equaled 1 if the individual self-reported being hospitalized in the previous 12 months before the date of the interview and 0 otherwise; this variable was based on the following CFPS question: ‘Have you been hospitalized in the past twelve months?’

### Independent variables

First, all adults were divided into three mutually exclusive smoking-status groups: never smokers, current smokers, and former smokers. Each adult was asked, ‘Have you smoked cigarettes in the past month?’. If the adult reported ‘yes’, then the respondent was categorized as a current smoker. The respondents who reported ‘no’ were then asked, ‘Have you ever smoked?’. If the adult reported ‘yes’, then the adult was considered a former smoker. If the adult answered ‘no’ to both questions, then the adult was categorized as a never smoker. Second, regular drinking was defined as a dummy variable. The CFPS question supporting this variable is as follows: ‘Have you often drunk alcohol more than 3 times a week?’. The adults who answered ‘yes’ were coded as 1, and those who answered ‘no’ were coded as 0. Third, the respondents were classified as unhealthy weight based on their BMI. The BMI was calculated based on the following CFPS questions: ‘What is your height (centimeters)?’ and ‘What is your weight (0.5 kilograms)?’. For further analysis, the BMI was categorized into the following four groups based on the World Health Organization Asian BMI cutoff points: underweight (< 18.5), healthy weight (18.5–22.9), overweight (23.0–27.5), and obese (> = 27.5) [36].

Lastly, to control for the possible effect of confounding factors, the control variables were selected based on the emerging behavioral model of health services use, and this model requires longitudinal study designs [37]. The predisposing factors in this study were age, gender, marital status, urban residency, educational attainment, and employment status. The enabling factors included household income and medical insurance coverage. The perceived need factors were represented by self-reported health status and chronic disease. The definitions of the control variables are provided in Table 1.

**Table 1:** Definitions of the control variables

Variable	Description
Age group	
16–24	Coded 1 if the individual is 16–24 years old and 0 otherwise
25–64	Coded 1 if the individual is 25–64 years old and 0 otherwise
> =65	Coded 1 if the individual is > = 65 years old and otherwise
Male	Coded 1 if the individual is male and 0 if female
Educational attainment	
Illiteracy	Coded 1 if the individual is illiterate or semiliterate and 0 otherwise
Elementary school	Coded 1 if the individual attended elementary school and 0 otherwise
Middle school	Coded 1 if the individual graduated from middle school and 0 otherwise
High school	Coded 1 if the individual graduated from high school and 0 otherwise
Above high school	Coded 1 if the individual graduated from above high school and 0 otherwise
Married	Coded 1 if the individual is married and 0 otherwise
Urban residency	Coded 1 if the individual is an urban resident and 0 if a rural resident
Medical insurance coverage	Coded 1 if the individual is enrolled in a medical insurance scheme and 0 otherwise
Household income	Net household income (10,000 yuan); the CFPS measured comparable household income between 2010 to 2018
Employed	Coded 1 if the individual reported participating in an agricultural job, working for wages for an employer, or working for oneself rather than an employer; coded 0 if the individual reported being a temporary worker, retired, unemployed, or a student;
Health status	
Poor	Coded 1 if the individual reported his or her health status as being poor and 0 otherwise
Fair	Coded 1 if the individual reported his or her health status as being fair and 0 otherwise
Good	Coded 1 if the individual reported his or her heath status as being good, very good, or excellent and 0 otherwise
Chronic diseases	Coded 1 if the individual has had doctor-diagnosed chronic diseases in the past 6 months and 0 otherwise

Note: 1000 yuan is equal to approximately $150 USD

### Statistical analysis

Panel data, also known as longitudinal data in epidemiology, are a dataset in which observations of multiple subjects are collected over time. Panel data can be used to control for time-constant unobserved heterogeneity and omitted time-varying variables [34]. Based on a five-period balanced panel dataset, the present study estimated the impact of smoking, regular drinking, and unhealthy weight on health care utilization by employing logistic regression models and assumed that there is an unobserved latent variable ${y}_{it}^{\ast }$ that is linked to the observed binary response variable (health care utilization).

$${y}_{it}^{\ast }={x}_{it}^{\prime}\alpha+{\beta}_1{Csmoking}_{it}+{\beta}_2{Fsmoking}_{it}+{\beta}_3{Drinking}_{it}+{\beta}_4{\mathrm{Uweight}}_{it}+{\beta}_5{\mathrm{Oweight}}_{it}+{\beta}_6{\mathrm{Obese}}_{it}+{\mu}_i+{\varepsilon}_{it},\kern0.5em i=1,\dots, n,t=1,\dots, {T}_i$$

${x}_{it}^{\prime }$ is a vector of confounders including age, gender, marital status, urban residency, educational attainment, employment status, household income, medical insurance coverage, self-reported health status, and chronic disease. *Csmoking*_*it*_, *Fsmoking*, *Drinking*_*it*_, Uweight_*it*_, Oweight_*it*_, and Obese_*it*_ are dummy variables that are defined as current smoker, former smoker, regular drinker, underweight, overweight, and obese. *μ*_*i*_ is the unobserved and individual specific heterogeneity, and *ε*_*it*_ is a time-varying error term.

There is a binary variable *y*_*it*_ where

$${y}_{it}=1\ if\ {y}_{it}^{\ast }>0, and\ 0\ therwise$$

where *y*_*it*_ =1 indicates that the individual visited outpatient care (or was hospitalized).

Thus, the probability that *y*_1*it*_ =1 is as follows:

$$P\left({y}_{it}=1\ \right|\ {x}_{it},\beta, {\mu}_i\left)=P\left({y}_{it}^{\ast }>0\ \right|\ {x}_{it},\beta, {\mu}_i\right)$$

$$=P\left({x}_{it}^{\prime}\beta +{\mu}_i+{e}_{it}>0\ \right|\ {x}_{it},\beta, {\mu}_i\Big)$$

$$=P\left({\varepsilon}_{it}<{\mu}_i+{x}_{it}^{\prime}\beta\ \right|\ {x}_{it},\beta, {\mu}_i\Big)$$

$$=F\left({\mu}_i+{x}_{it}^{\prime}\beta \right)$$

The study further assumes that the error term *ε*_*it*_ is logistically distributed, and we arrive at the following logistic regression model:

$$P\left(\left({y}_{it}=1\ \right|\ {x}_{it},\beta, {\mu}_i\right)=\frac{{\mathit{\exp}}^{\mu_i+{x}_{it}^{\prime}\beta }}{1+{\mathit{\exp}}^{\mu_i+{x}_{it}^{\prime}\beta }}$$

$$P\left(\left({y}_{it}=0\ \right|\ {x}_{it},\beta, {\mu}_i\right)=\frac{1}{1+{\mathit{\exp}}^{\mu_i+{x}_{it}^{\prime}\beta }}$$

Furthermore, the assumption that unobserved heterogeneity *μ*_*i*_ is uncorrelated with *x*_*it*_ produces a random effects logistic model. However, when *μ*_*i*_ is correlated with *x*_*it*_, then it is called a fixed effects logistic model [30, 34].

In the first step in the analysis, pooled logistic regression can increase the sample size and be a starting point. Subsequently, this study treats the data as having a panel structure and chooses between the fixed effects and random effects logistic models. In this study, a possible unobserved variable is health literacy, which is correlated with the time-varying explanatory variables in the model (e.g., smoking, regular drinking, or unhealthy weight). The fixed effects logistic model can control for omitted variable bias at the unit level. With such correlated heterogeneity and Hausman’s specification test results, the fixed effects logistic model should be preferred over the random effects logistic model. However, when estimating the fixed effects logistic model, many pieces of information are lost. Therefore, the random effects logistic model is also presented in this study. The results are presented as odds ratios (ORs) along with 95% confidence intervals (CIs). All statistical analyses are conducted employing the STATA 15 statistical software package.

## Results

A descriptive summary of the selected variables over time is displayed in Table 2. The proportion of visits to outpatient care increased from 18.37% in 2010 to 27.01% in 2018. Hospitalization rates sharply increased from 7.33% in 2010 to 15.05% in 2018. These results indicate an increasing tendency for health care utilization. Approximately one in three respondents were current smokers from 2010 to 2018. Moreover, the proportions of former smokers rose from 6.16% in 2010 to 12.22% in 2018. Approximately 17.0% of the respondents were regular drinkers from 2010 to 2018. In 2010, the proportions of overweight and obese individuals were 35.24 and 7.41%, respectively; in 2018, these proportions increased to 41.84 and 12.13%, respectively.

**Table 2:** Description of the selected variables over five waves

	Wave 1	Wave 2	Wave 3	Wave 4	Wave 5
	2010	2012	2014	2016	2018
Outpatient care utilization (%)	18.37	20.90	24.19	24.03	27.01
Inpatient care utilization (%)	7.33	8.60	10.95	12.94	15.05
Smoking status (%)					
Never smokers	62.70	60.50	59.52	58.93	58.52
Current smokers	31.14	30.58	29.87	28.58	29.26
Former smokers	6.16	8.92	10.61	12.49	12.22
Regular drinking (%)	16.77	16.99	17.04	16.46	16.88
Body mass index (%)					
Underweight	7.63	7.88	6.81	6.66	5.53
Healthy weight	49.73	46.70	43.85	42.43	40.50
Overweight	35.24	36.26	39.10	39.42	41.84
Obese	7.41	9.16	10.24	11.50	12.13
Observations	12,652	12,652	12,652	12,652	12,652

Table 3 presents the regression analysis results of the pooled logistic, random effects logistic, and fixed effects logistic models (outpatient care utilization). Since the likelihood ratio (LR) test showed highly significant test statistics (LR = 1086.53) for the random effects logistic model, the unobserved heterogeneity was significant, and panel estimation methods were needed in this study. Hausman’s specification test showed a test statistic *χ* ^2^(20) =1268.40, which was significant at the 1% level. Hence, based on the Hausman test, fixed effects estimates should be preferred over random effects estimates.

**Table 3:** Logistic regression analysis of outpatient care utilization

	Pooled logistic	Random effects logistic	Fixed effects logistic
	(i)	(ii)	(iii)
	Odds Ratios (95% CI)	Odds Ratios (95% CI)	Odds Ratios (95% CI)
Smoking status			
Current smoker	1.12 (1.03–1.21)	1.16 (1.06–1.27)	1.88 (1.42–2.51)
Former smoker	1.12 (1.01–1.24)	1.20 (1.07–1.33)	2.03 (1.51–2.74)
Never smoker (ref.)	1.00	1.00	1.00
Regular drinking	0.82 (0.76–0.88)	0.80 (0.74–0.87)	0.83 (0.75–0.93)
BMI groups			
Healthy weight (ref.)	1.00	1.00	1.00
Underweight	1.11 (1.02–1.21)	1.13 (1.03–1.25)	1.09 (0.96–1.24)
Overweight	0.96 (0.91–1.01)	0.96 (0.91–1.02)	1.02 (0.93–1.10)
Obese	0.99 (0.91–1.08)	1.03 (0.94–1.13)	1.26 (1.08–1.46)
Age group			
16–24 (ref.)	1.00	1.00	1.00
25–64	1.55 (1.32–1.83)	1.58 (1.32–1.89)	1.17 (0.91–1.50)
> =65	1.98 (1.67–2.35)	2.20(1.83–2.66)	1.69 (1.28–2.23)
Male	0.71 (0.66–0.77)	0.65 (0.60–0.71)	–
Educational attainment			
Illiteracy (ref.)	1.00	1.00	1.00
Elementary school	0.88 (0.82–0.94)	0.85 (0.79–0.92)	0.98 (0.82–1.17)
Middle school	0.85 (0.80–0.91)	0.82 (0.76–0.88)	1.04 (0.81–1.33)
High school	0.79 (0.72–0.87)	0.75 (0.68–0.82)	1.16 (0.81–1.65)
Above high school	0.76 (0.68–0.86)	0.70 (0.62–0.80)	1.20 (0.77–1.87)
Married	0.96 (0.89–1.04)	0.95 (0.86–1.04)	0.82 (0.69–0.98)
Urban residency	0.90 (0.86–0.95)	0.91 (0.86–0.97)	1.26 (1.09–1.45)
Medical insurance coverage	1.18 (1.09–1.28)	1.19 (1.09–1.30)	1.12 (1.01–1.23)
Household income	1.00 (0.99–1.00)	1.00 (1.00–1.01)	1.01 (1.00–1.02)
Employed	1.14 (1.08–1.21)	1.18 (1.11–1.25)	1.23 (1.13–1.33)
Health status			
Poor	3.07 (2.89–3.26)	3.44 (3.21–3.68)	2.49 (2.30–2.69)
Fair (ref.)	1.00	1.00	1.00
Good	0.46 (0.43–0.48)	0.44 (0.42–0.47)	0.57 (0.53–0.61)
Chronic disease	3.09 (2.93–3.26)	3.23 (3.06–3.42)	2.21 (2.07–2.35)
Constant	0.18 (0.15–0.22)	0.15 (0.12–0.18)	–
Observations	63,260	63,260	34,520

Note: Likelihood ratio test in random effects logistic model: *LR* =1086.53, *p* < 0.0001

Hausman’s specification test is not significant at the 5% level: *χ* ^2^(20) =1268.40, *p* < 0.0001

The results of the logistic regression analysis are shown in Table 3 as ORs. Column (iii) of Table 3 presents the factors associated with outpatient care utilization using the fixed effects logistic model. The results reveal that smoking status is associated with the use of outpatient care. Current and former smokers were approximately 1.9 times and 2.0 times more likely to use outpatient care than those who never smoked, respectively (OR = 1.88, *p* < 0.05; OR = 2.03, *p* < 0.05). People who regularly drank alcohol were 17% less likely to use outpatient care than nonregular drinkers (OR = 0.83, *p* < 0.05). Obese people were approximately 1.3 times more likely to use outpatient care than healthy weight people (OR = 1.26, *p* < 0.05).

Irrespective of the estimation method, current and former smokers showed an increased probability of using outpatient care compared to those who had never smoked. In contrast, regular drinkers were less likely to use outpatient care than nondrinkers (see Columns (i)-(iii) of Table 3).

Table 4 shows the regression analysis results of the pooled logistic, random effects logistic, and fixed effects logistic models (inpatient care utilization). Based on the LR test and Hausman’s specification test, fixed effects estimation was the preferred method for the panel data in this study.

**Table 4:** Logistic regression analysis of inpatient care utilization

	Pooled logistic	Random effects logistic	Fixed effects logistic
	(i)	(ii)	(iii)
	Odds Ratios (95% CI)	Odds Ratios (95% CI)	Odds Ratios (95% CI)
Smoking status			
Current smoker	1.00 (0.91–1.11)	1.02 (0.91–1.13)	1.42 (0.99–2.05)
Former smoker	1.60 (1.43–1.79)	1.78 (1.58–2.01)	2.98 (2.05–4.35)
Never smoker (ref.)	1.00	1.00	1.00
Regular drinking	0.79 (0.73–0.87)	0.76 (0.69–0.84)	0.68 (0.59–0.77)
BMI groups			
Healthy weight (ref.)	1.00	1.00	1.00
Underweight	0.96 (0.85–1.07)	0.95 (0.84–1.08)	0.96 (0.82–1.14)
Overweight	1.01 (0.95–1.08)	1.03 (0.96–1.10)	1.11 (1.00–1.23)
Obese	1.05 (0.95–1.17)	1.07 (0.96–1.19)	1.18 (0.98–1.41)
Age group			
16–24 (ref.)	1.00	1.00	1.00
25–64	1.10 (0.89–1.35)	1.08 (0.87–1.34)	0.87 (0.65–1.18)
> =65	2.05 (1.65–2.54)	2.17 (1.74–2.71)	1.53 (1.10–2.12)
Male	0.92 (0.84–1.01)	0.89 (0.80–0.98)	–
Educational attainment			
Illiteracy (ref.)	1.00	1.00	1.00
Elementary school	0.89 (0.82–0.97)	0.88 (0.80–0.96)	1.14 (0.92–1.42)
Middle school	0.87 (0.80–0.95)	0.85 (0.78–0.93)	1.07 (0.78–1.46)
High school	0.99 (0.89–1.11)	0.97 (0.87–1.09)	1.31 (0.83–2.06)
Above high school	0.83 (0.72–0.97)	0.78 (0.67–0.92)	1.10 (0.60–2.02)
Married	1.01 (0.91–1.13)	1.01 (0.91–1.13)	0.97 (0.78–1.19)
Urban residency	1.13 (1.06–1.21)	1.15 (1.07–1.23)	1.29 (1.07–1.54)
Medical insurance coverage	1.68 (1.50–1.88)	1.70 (1.50–1.91)	1.45 (1.27–1.66)
Household income	1.00 (0.99–1.00)	1.00 (1.00–1.01)	1.01 (1.00–1.01)
Employed	0.92 (0.86–0.98)	0.92 (0.86–0.99)	0.99 (0.90–1.09)
Health status			
Poor	2.22 (2.05–2.40)	2.38 (2.19–2.59)	1.94 (1.75–2.14)
Fair (ref.)	1.00	1.00	1.00
Good	0.70 (0.65–0.75)	0.69 (0.64–0.74)	0.79 (0.72–0.86)
Chronic disease	2.81 (2.65–2.99)	2.99 (2.80–3.19)	2.31 (2.14–2.50)
Constant	0.05 (0.04–0.06)	0.04 (0.03–0.05)	–
Observations	63,260	63,260	22,650

Note: Likelihood ratio test in random effects logistic model: *LR* =622.00, *p* < 0.0001

Hausman’s specification test is not significant at the 5% level: *χ* ^2^(20) =303.22, *p* < 0.0001

Column (iii) of Table 4 presents the factors associated with inpatient care utilization using the fixed effects logistic model. The results show that compared to those who never smoked, for current and former smokers, the odds of being hospitalized increased 42.2 and 198.2%, respectively (OR = 1.42; *p* < 0.1, OR = 2.98; *p* < 0.05). Regular drinking showed 32% lower odds of being hospitalized than nonregular drinking (OR = 0.68, *p* < 0.05). Compared to healthy weight people, overweight and obese people were more likely to be hospitalized (OR = 1.11; *p* < 0.1, OR = 1.18; *p* < 0.1, respectively).

Irrespective of the estimation method, former smokers showed an increased probability of obtaining inpatient care compared to never smokers. In contrast, regular drinkers were less likely to be hospitalized than nonregular drinkers (see Columns (i)-(iii) of Table 4).

## Discussion

The aim of this study was to ascertain the effects of smoking, regular drinking, and unhealthy weight on health care utilization in China. First, this study found that among Chinese adults, current and former smokers are more likely to use outpatient and inpatient care than those who have never smoked. The explanation is straightforward. Smoking has adverse health effects and causes acute and chronic diseases; therefore, smokers may require more health care utilization. Moreover, the current study found that compared to current smokers, former smokers have increased odds of using outpatient and inpatient care. Former smokers use more health care services simply because many of them have stopped smoking due to health concerns and serious illnesses they have experienced [38, 39].

Previous studies have found that smokers commonly bear higher health care costs than those who have never smoked [40–42]. China has successfully achieved universal health insurance coverage, with approximately 95% of the population being insured in 2011. However, the sources of financing health insurance plans are no different between smokers and nonsmokers. Therefore, people who have never smoked may subsidize the health care costs of smokers.

Second, the current study found that among Chinese adults, people who regularly drink alcohol are less likely to use outpatient and inpatient care than nonregular drinkers. This result is consistent with findings for American drinkers [22], rural Liberian drinkers [23], and German drinkers [43]. Three possible reasons may explain the inverse relationship between regular drinking and health care utilization. First, people who regularly drink alcohol may not care about their health status or may be risk-tolerant individuals [25, 44]. Second, the adverse health consequences of drinking may appear several years later. Third, alcohol use was measured by a dichotomous variable (regular drinking) in this study. The CFPS lacks information on the frequency and quantity of drinking, which makes it challenging to evaluate drinking severity and to distinguish between heavy drinking and drinking in moderation. Heavy drinking increases one’s risk for adverse health events [45]. Lastly, alcohol is frequently used by men in China, and Chinese cultural norms encourage social drinking, especially with friends and family [46]. Nondrinkers in such a cultural or social situation may have alcohol allergies and intolerance; hence, they may use more health care services [47]. Notably, people who regularly drink alcohol experience omitted or delayed health care, which leads to serious health problems and higher health care costs for society [25].

Third, the present study found that among Chinese adults, obese people are more likely to use outpatient and inpatient care than healthy weight people. In addition, overweight people are more likely to be hospitalized than healthy weight people. Higher morbidity associated with being overweight and obese has been observed for hypertension, diabetes, coronary heart disease, stroke, and cancers [48]. Hence, a higher level of health care utilization may be needed to treat these conditions.

In the context of universal healthcare coverage and the aging population in China, this analysis has identified several approaches to help health authorities better ground their strategies. First, local communities and primary care facilities should consider promoting health education programs for smokers and improving smokers’ understanding of the hazards of smoking. Moreover, the Chinese government should consider raising taxes on tobacco, which will not only help to reduce the prevalence of smoking but also increase revenues, which can be used to financially sustain public health insurance plans. Second, local communities and primary care facilities should develop outreach and educational programs to minimize the adverse health consequences for regular drinkers. In addition, the Chinese government should conduct screenings and brief advice programs delivered by primary-level care, thus reducing the burden of diseases due to regular drinking. Third, local communities should develop local physical activity intervention strategies for people with unhealthy weight. Furthermore, the Chinese government should promote a national public education campaign to encourage overweight and obese people to maintain a healthy weight, thereby improving their health status and considerably lowering health care costs.

This study has several limitations that should be emphasized. First, the CFPS survey provides information only on the most recent outpatient service utilized in the previous 2 weeks. Measuring outpatient care utilization using the most recent outpatient visits within the last 2 weeks may underestimate the utilization of outpatient care among Chinese adults. Moreover, the frequency of drinking and the amount of alcohol used were unavailable. Measuring alcohol use with a dichotomous variable (regular drinking) may bias the results. Second, self-reported smoking, drinking, height, and weight were used in this study and thus share the limitations of all self-reported data, namely, recall bias and unreliability under pressure. Third, this study could not exclude ex-drinkers from the group of nonregular drinkers. If ex-drinkers stopped drinking due to severe illnesses, this might affect the inverse association between regular drinking and health care utilization. Fourth, when the respondents without changes in their health care utilization decisions across all five waves did not contribute to the likelihood, the fixed effects model lost many pieces of information. For example, when estimating a fixed effects model, whenever the respondents use (or do not use) outpatient (or inpatient) care across all five waves, by definition, there will be no variation over time, and these respondents will be dropped from the empirical estimation. Therefore, the estimated results will be less precise and have larger standard errors. Finally, although the current study was adjusted for a wide variety of demographic and socioeconomic variables, it is possible that unmeasured confounders may explain the current findings.

## Conclusions

The purpose of this study is to empirically ascertain the effects of smoking, regular drinking, and unhealthy weight on health care utilization in China. The empirical findings suggest that among Chinese adults, current and former smokers are more likely to use health care than those who have never smoked. Moreover, obese and overweight people are more likely to use health care than healthy weight people. These results may have important implications that support the government in making health care resource allocation decisions.

## Data Availability

The datasets generated and/or analyzed during the current study are available in the Peking University Open Research Data Platform repository, https://opendata.pku.edu.cn/dataset.xhtml?persistentId=doi:10.18170/DVN/45LCSO.

## Abbreviations

CFPS: China Family Panel Studies
BMI: Body mass index
LR: Likelihood ratio
OR: Odds ratio
